# The discovery of actin: “to see what everyone else has seen, and to think what nobody has thought”*

**DOI:** 10.1007/s10974-019-09515-z

**Published:** 2019-05-15

**Authors:** Beáta Bugyi, Miklós Kellermayer

**Affiliations:** 1 Department of Biophysics, Medical School, University of Pécs, Szigeti str. 12, Pécs, 7624 Hungary; 2 Department of Biophysics and Radiation Biology, Faculty of Medicine, Semmelweis University, Tűzoltó str. 37-47, Budapest, IX 1428 Hungary

**Keywords:** Actin, Actomyosin, Muscle contraction, Albert Szent-Györgyi, Ilona Banga, Brúnó F. Straub

## Abstract

Actin is among the most highly abundant and ubiquitous proteins in eukaryotic cells. The structure, dynamics and functional diversity of actin have continued to mesmerise cell and molecular biologists, biophysicists and physiologists for more than three quarters of a century. The discovery and initial characterization of actin, which took place in the laboratory of Albert Szent-Györgyi by Ilona Banga and Brúnó F. Straub during the second world war in Hungary, is a remarkable and inspiring moment in the history of science. Many of the early thoughts and ideas on the properties and functions of actin and particularly actomyosin, which are referred to in this short historical overview, resonate freshly even today.

## Introduction

The discovery of actin in the Albert Szent-Györgyi laboratory in 1942 represents one of the most important findings in all of biomedical sciences. It coincides with the beginnings of serious studies in muscle biochemistry, most significantly because it showed that the interaction of actin with myosin was involved in contraction. Later it turned out that actin is important not only in muscle, but it is an integral constituent of the non-muscle eukaryotic cytoskeleton and contributes to a whole array of cellular processes. The subsequent emergence of a huge inventory of actin-binding proteins further underscores actin’s multi-faceted roles [reviewed in (Bugyi and Carlier 2010; Blanchoin et al. 2014; Pollard 2016; Carlier and Shekhar 2017)]. Over the years, actin’s versatility has been extended by the realization that it is an essential component of the cell nucleus and contributes to nucleoskeleton function, and that actin homologues also exist in prokaryotes [reviewed in (Clark and Merriam 1977; Cabeen and Jacobs-Wagner 2010; Jiang et al. 2016; Kristo et al. 2016; Viita and Vartiainen 2017)]. Furthermore, actin inspired engineers, mathematicians and computer scientists to mimic its intrinsic properties to assemble electrical connections (Galland et al. 2013) or to use the actomyosin machinery for parallel computation in nanofabricated “biocomputers” (Nicolau et al. 2016).

**Fig. 1:**
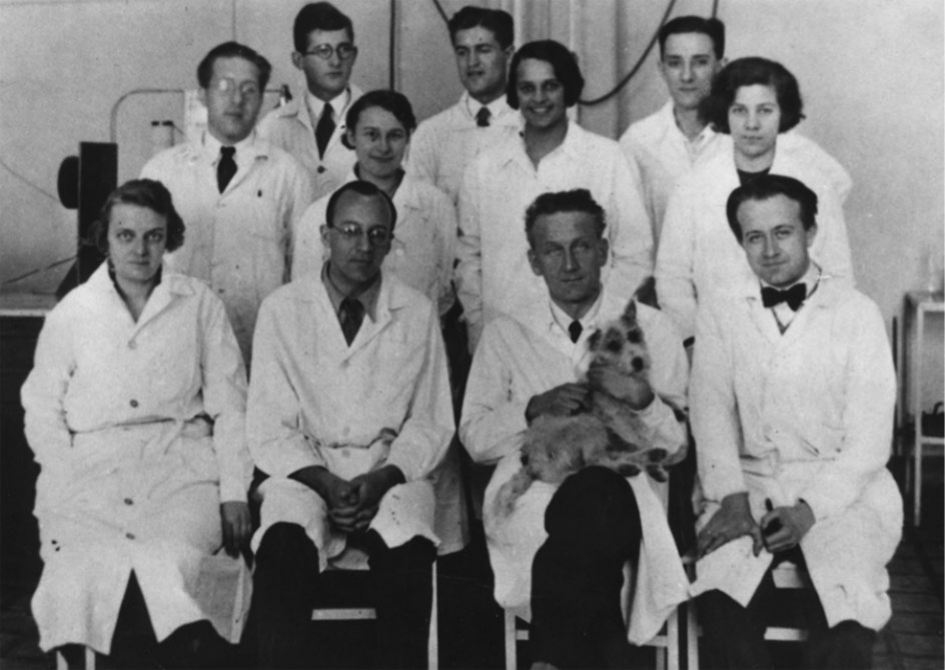
Albert Szent-Györgyi’s research group (Szeged 1933) (Fésus 2013). First row: Margaret Zétényi (secretary), Béla Gozsi (pharmacist), Albert Szent-Györgyi, Ernő Annau (biomedical, biochemist); second row: Joseph L. Svirbely (guest researcher), Ilona Banga, Nelli Szent-Györgyi (Szent-Györgyi’s daughter), Edit Joó (laboratory assistant); third row: Brúnó F. Straub, Kálmán Laki, Sándor Szalay (physicist)

The story of actin began in the early 1940’s in Albert Szent-Györgyi’s laboratory at the Institute of Medical Chemistry in the south Hungarian city of Szeged. The lab was equipped with simple instrumentation that included centrifuges, Ostwald viscometers and polarizing filters to measure flow birefringence. The atmosphere was pervaded by World War II. Inspired by the intrinsic pursuit to understand the secrets of living matter, this highly motivated group of scientists (Fig. 1) made fundamental discoveries about the mechanisms of muscle contraction that can be summarized in the following:

1. A novel muscle protein was discovered and isolated from muscle tissues, named actin.
2. Actin exists in two different forms, named G-actin and F-actin, which are interconverted upon reversible polymerization in a salt-dependent manner. Actin possesses ATPase activity that is connected to its polymerization.
3. The contractile behavior of muscle essentially originates from the interaction of actin, myosin and ATP.

In the wartime years it was difficult to publish the results internationally. The findings of the lab first appeared in the three volumes of the *Studies from the Institute of Medical Chemistry University Szeged,* printed in Hungary between 1941 and 1943. Szent-Györgyi, recognizing the significance of the findings, was afraid that the discoveries might remain hidden from the scientific community; therefore, he made an enormous effort to get them published abroad in German and Swedish journals (Szent-Györgyi 1942a, b, c; Szent-Györgyi 1945). A facsimile reproduction of the original work was published recently in *Muscle Contraction: a Hungarian Perspective* along with biographical chapters of eminent Hungarian muscle researchers^1^ (Kellermayer 2018). The *Studies* are remarkable not only for their historical importance but also for their scientific rigor, clear thinking and logical reasoning.

## Discovery of actin: a *“compound”* that changes *“the qualities of myosin itself*” (Banga and Szent-Györgyi 1942)

Albert Szent-Györgyi was awarded the Nobel Prize for Physiology and Medicine in 1937 *“for his discoveries in connection with the biological combustion processes, with special reference to vitamin C and the catalysis of fumaric acid”*. His innate impulse to get *“closer to the understanding of life”* motivated him to *“attack some more complex biological process”* (Szent-Györgyi 1963, 2004). According to his views *“it does not matter which material we choose for our study of life, be it grass or muscle, virus or brain. If we only dig deep enough we always arrive at the center, the basic principles on which life was built and due to which it still goes on.”* (Szent-Györgyi 1951). Inspired by *“its violent physical, chemical, and dimensional changes”*, he considered that *“muscle is an ideal material to study”* (Szent-Györgyi 1963). By that time myosin was already known as an integral muscle protein and an enzyme that possesses ATPase activity (Kühne 1864; Engelhardt and Ljubimowa 1939). *“It is evident, then, that we need to understand myosin if we want to understand contraction.”* exclaimed Szent-Györgyi in the opening chapter of *Studies* Vol. 1 (Banga and Szent-Györgyi 1942).

Albert Szent-Györgyi and Ilona Banga prepared myosin from rabbit skeletal muscle following the classic 20-min extraction in high concentration of potassium chloride.^2^ One day Banga finished the extraction late in the afternoon and decided to leave the myosin suspension overnight in the cold room (Banga 1983). Next morning she observed with surprise that the myosin extract became a jelly-like, viscous suspension as compared to the original 20-min muscle extract. It was shown that *“myosin can be obtained from muscle in two different forms”* depending on the exposure time (Banga and Szent-Györgyi 1942); the 20-min and 24-h extracts were named myosin A and B, respectively. They sought to understand the *“relation of the two substances … myosin A and B and what is the nature of the A → B transformation”* (Szent-Györgyi 1942a, b, c). Viscometry analysis corroborated that the rheological properties of myosin A and B differ; myosin B had higher viscosity than myosin A. Importantly, the viscosity of myosin B decreased markedly upon the addition of ATP, while that of myosin A was unaffected. They noticed that when ATP was depleted, myosin B *“gelatinised again”* (Banga and Szent-Györgyi 1942). Altogether, these results clearly indicated that the effect of ATP was reversible and specific for myosin B. To quantitatively express the *“activity of myosin”*, Szent-Györgyi introduced *“the fall of viscosity on addition of ATP”* (Banga and Szent-Györgyi 1942). The different *activity* of myosin A and B was interpreted as the *“change in the qualities of myosin itself”,* and it was proposed that *“myosin B is a stoichiometric compound of myosin A and another substance”* (Banga and Szent-Györgyi 1942; Szent-Györgyi 1942a, b, c). Since the newly identified compound influenced the *activity* of myosin, it was named *actin*. Meanwhile, Brúnó F. Straub was able to separate actin from myosin. He employed two strategies based on fractional extraction of myosin and actin. First, as a starting material he used the 24-h extract myosin B. After washing it with distilled water and centrifuging, the precipitate was dehydrated by acetone that denatures myosin and makes it insoluble. Actin was extracted from the acetone-dried powder with distilled water. Alternatively, he brought myosin A into solution by applying the 20-min extraction of rabbit muscle. The suspension was centrifuged, and thereby a large fraction of myosin could be removed with the supernatant. Subsequently, the pellet was washed with distilled water and centrifuged. The residue was treated with acetone to remove the remaining myosin content. The dried acetone power was extracted with distilled water, and undissolved components were clarified by centrifugation to obtain actin (Straub 1942). Upon mixing the purified actin with myosin A, the *activity* of myosin B (i.e. the fall of its viscosity upon the addition of ATP) was reconstituted,^3^ demonstrating that myosin B is *“a compound of myosin and actin”* (Straub 1942). The closing chapter of *Studies* Vol I sets the nomenclature: *“we will call this other compound actin and the myosin*-*actin complex will be called acto*-*myosin”* (Szent-Györgyi 1942a, b, c).

## Uncovering actin’s intrinsic biochemical nature

Unequivocally, the discovery of actin as a novel muscle protein lent strong motivation to the Szent-Györgyi group to understand its intrinsic biochemical nature. The route was opened by Straub by developing the preparation of acetone-dried muscle powder and succeeding in isolating actin from it (Straub 1942, 1943). It is noteworthy that Straub’s protocol is still the basis of the method to purify actin from muscle sources. Straub’s experiments showed that actin can exist in two different forms depending on the biochemical milieu. Distilled water extraction of the acetone-dried powder resulted in a protein that did not change the viscosity of myosin when added to it, and the properties of this actomyosin were not affected by ATP, either. Thus, it did not influence the *activity* of myosin; he called it *“inactive actin”* (Straub 1943). By contrast, when the inactive actin was treated with salts (0.1 M KCl for 5 min at 22 °C) before mixing it with myosin, it could increase the viscosity of myosin in an ATP-sensitive manner; this actin was called *“active actin”* (Straub 1943). Straub recognized that the two forms of actin were not only functionally different but had different geometries, as well; *“viscosity of an inactive actin solution is very low… thus, … inactive actin is classed among the globular proteins”*, while *“active actin … has a very high viscosity which equals to the viscosity of polymer substances…it has therefore an extremely asymmetrical molecule of considerable length”* classed as a *“fibrous one”* (Straub 1943). The transformation between the two forms of actin, i.e. *“actin activation”,* was proposed to be the result of polymerization that *“must be ascribed to ions”* (Straub 1943; Feuer et al. 1948). The globular and fibrous geometries of actin were named G-, and F-actin, respectively, by Szent-Györgyi (Szent-Györgyi 1945). Considering the above findings and that Straub’s viscometry measurements were performed in high salt conditions (0.6 M KCl), the absence of polymerization and therefore the lack of myosin activation *“is somewhat surprising”*, as Straub wondered himself (Straub 1943). He reasoned that *“It follows therefore that a combination must have taken place between inactive actin and myosin and that this combination prevents the inactive actin from being activated.”* (Straub 1943). Straub supported this experimentally in two ways. He added ATP to the mixture of *inactive* actin and myosin and waited until the nucleotide was split. The subsequent addition of fresh ATP resulted in a decrease in the viscosity of the actomyosin solution. Straub concluded that *“the activation of inactive actin in the experiment, which contained ATP, is no doubt due to the fact that ATP has split the complex of inactive actomyosin, setting free the actin thus making it accessible to the activating effect of salts.”* Secondly, he found that *“if an excess inactive actin is added to the myosin … it will become partially activated as the myosin will not be able to bind it completely”*. These observations indicating that myosin can interact with the monomeric form of actin which is weakened by ATP were further corroborated and comprehensively investigated in later studies (Valentin-Ranc et al. 1991; Blanchoin et al. 1995).

Apart from the results presented in the *Studies*, subsequent work performed at the end of 1940’s by Straub’s laboratory indicated that ATP is a functional group of actin, and that the protein also binds divalent cations. They proposed *“that actin is a coordination complex in which Ca forms a bond both with the protein and with the ATP”* (Feuer et al. 1948; Straub and Feuer 1950). It was shown that *“the polymerization of actin is connected with the simultaneous formation of ADP and inorganic phosphate from the ATP present in actin. In other words, globular actin is ATP*-*actin; ADP*-*actin, if formed, is in the fibrous form, i.e. polymerized”* (Feuer et al. 1948; Straub and Feuer 1950). They also found that *“when dialysed against ATP”* in salt-free environment, *“fibrous actin depolymerizes and acquires again bound ATP”*, showing that actin polymerization is a reversible process (Straub and Feuer 1950).

It is worth noting that Straub’s lab could not reverse polymerization by alternative means, except dialysis. As they noted intuitively, *“one great problem of actin chemistry remains still unsolved, and that is the mode of physiological depolymerization”* (Straub and Feuer 1950). It is now established that the cellular control of actin disassembly is achieved by the action of different actin-binding proteins including ADF/cofilin and gelsolin family proteins, first identified in the 1980’s. The physiological and mechanistic understanding of the disassembly of actin structures is still one of particular interest in actin biology, biochemistry and biophysics (Blanchoin et al. 2014; Ydenberg et al. 2015).

It is worth of noting that Kálmán Laki (Koloman Laki, as his name appears in publications, Fig. 1), even though a member of the Szent-Györgyi laboratory in the 1930s, did not work on muscle contraction but was interested in the biochemistry of hemostasis which led to the discovery of blood coagulation factor XIII (Laki 1943a, 1943b; Laki and Lorand 1948). Although he did not take part in the initial discovery of actin, later, as a special fellow at the NIH, he made valuable contributions to basic actin biochemistry, uncovering the interactions of actin with both muscle and non-muscle actin-binding proteins. For example, he contributed to the realization that the Straub-type actin preparation contains a significant portion of tropomyosin. He and his colleagues’ work suggested that “*the presence of tropomyosin in actin preparations is not accidental, but… there is an intimate interaction between actin and tropomyosin*”. Indeed, they were the first to provide evidence that tropomyosin is an F-actin binding protein (Laki et al. 1962). A resumé of his scientific achievements was published recently (Muszbek 2018).

## *“To see them contract for the first time”* (Szent-Györgyi 1963)

**Fig. 2:**
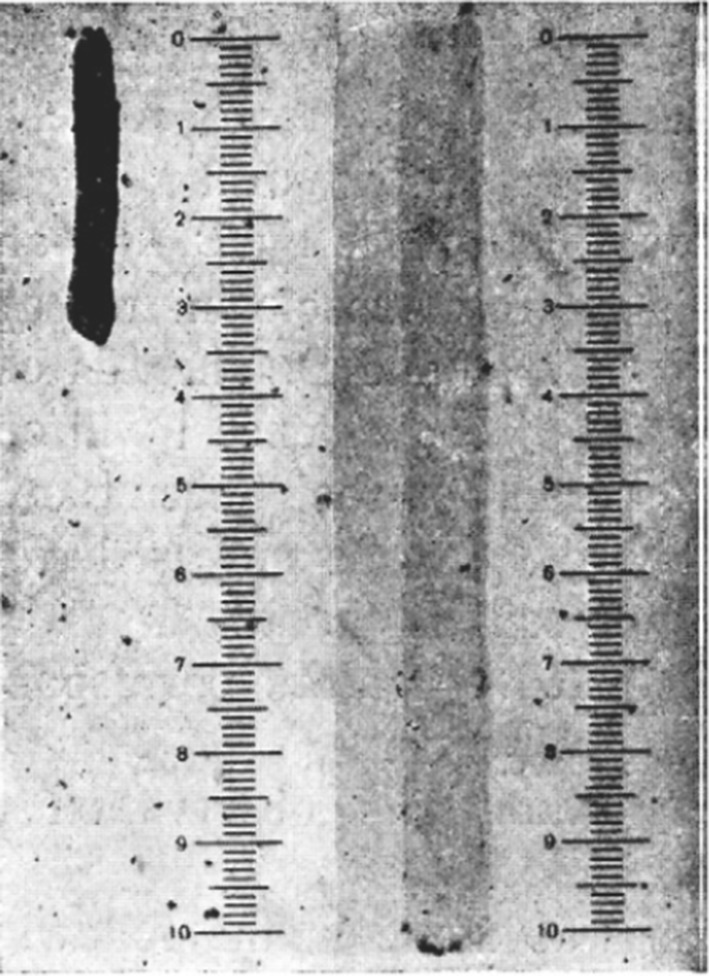
Contraction of actomyosin threads. Myosin B threads treated with water-extract of muscle (left) and in the absence of treatment (right) (Szent-Györgyi 1942a, b, c)

Szent-Györgyi was urged to further explore the behavior of actomyosin and its relation to ATP. He continued the studies on myosin threads, which were *“elongated pieces of myosin gels”* prepared from either myosin A or B (Gerendás 1942; Szent-Györgyi 1942a, b, c). By treating the threads with a water extract of muscle, myosin B threads quickly shortened in a process described by Szent-Györgyi as *“violent contraction”*. By contrast, no striking change was observed when myosin A was used (Fig. 2.) (Szent-Györgyi 1942a, b, c). As the first reconstruction of contractility, this was a groundbreaking finding. According to Szent-Györgyi, *“To see them contract for the first time, and to have reproduced in vitroone of the oldest signs of life, motion, was perhaps the most thrilling moment of my life.”* (Szent-Györgyi 1963).

The experiments made clear that the *“watery extract of the muscle contains thus something which causes violent contraction in myosin B threads”* (Szent-Györgyi 1942a, b, c). Since after overnight storage of the extract, it failed to stimulate contraction, Szent-Györgyi suspected that it was due to the depletion of ATP (Szent-Györgyi 1945, 1949, 1963). Indeed, addition of fresh ATP to the solution restored its ability to stimulate contraction. This *“little cookery”* showed that ATP is indispensable for the contractile behavior of actomyosin in vitro (Szent-Györgyi 1945, 1949, 1963). Szent-Györgyi proposed that *“muscle contraction was essentially an interaction of actomyosin and ATP”* (Szent-Györgyi 1963). Contractility, the biological functioning of muscle could be reconstituted from purified actin, myosin and ATP, providing the first biomimetic system of the contractile machinery.

Szent-Györgyi’s view was received with skepticism on the grounds that *“phenomena in actomyosin threads are fundamentally different from those in muscle”* (Szent-Györgyi 1963). To settle this criticism, Szent-Györgyi needed muscle fibres *“to be prepared, free of ATP, and made permeable to this substance, without destroying the actomyosin structure”* (Szent-Györgyi 1949). By extracting rabbit psoas muscle with glycerol at low temperature, Szent-Györgyi succeeded in obtaining muscle fibers free of ATP and with retained contractility (Szent-Györgyi 1949). The protocol established an experimental model for studying muscle contraction which is still in use today. Glycerol-extracted fibers contracted rapidly upon combining them with ATP. He demonstrated that *“if connected to the isometric lever, on addition of ATP they develop tension comparable in intensity to that developed by intact muscle on maximal excitation. If loaded they will also lift weights isotonically, similarly to intact muscle fibre bundles of similar dimensions”* (Szent-Györgyi 1949).

According to the theories of early muscle research *“contraction is a spontaneous process going hand*-*in*-*hand with a drop of free energy”* (Szent-Györgyi 1949). Therefore, *“contraction should occur spontaneously wherever the ATP*-*actomyosin system is present in a suitable ionic milieu, and the system should persist in the low*-*energy stable contracted state. This is actually what happens any time we add ATP to an actomyosin gel or to muscle extracted with water”*. Still it was considered puzzling that*” in the intact resting muscle, however, we find ATP in an active form, linked to actomyosin, but still the system does not contract—contraction being inhibited by some unknown mechanism. If we want the muscle to go over into the contracted state, we have to abolish this inhibition.”* (Szent-Györgyi 1949). Key missing pieces of the functional regulation of muscle contraction were revealed by subsequent pioneering studies establishing the classic *sliding filament* and *steric blocking theories* (Huxley and Niedergerke 1954; Huxley and Hanson 1954; Huxley 1957; Huxley and Simmons 1971; Haselgrove and Huxley 1973; Parry and Squire 1973; McKillop and Geeves 1993; Lehman et al. 1994; Lehrer and Geeves 1998; Geeves 2016; Lehman 2016, 2017).

## Discovery of actin: not a one-off accomplishment

For his work on muscle contraction Szent-Györgyi was awarded the Albert Lasker Basic Medical Research Award in 1954. It was only later revealed that he also received a second Nobel-Prize nomination in 1951, motivated by the discoveries in his laboratory on *“Muscular contraction and the role of myosin, actin and adenosine*-*triphosphate”.*^4^

Ilona Banga and Brúnó F. Straub had very different but successful later careers. Banga pursued scientific research throughout her life. In joint research with her husband (József Baló, pathology professor) on arteriosclerosis she studied the origin of fiber degradation in vein walls which led them to the discovery of the enzyme *elastase* produced by the pancreas (Balo and Banga 1949). Banga thus made exceptional contributions to three different areas; vitamin C and fumaric acid research (Nobel Prize awarded to Szent-Györgyi), muscle contraction (Szent-Györgyi nomination for a Nobel Prize) and arteriosclerosis research. In 1940, at an age of 34, she became the first female docent (associate professor) at the University of Szeged; in 1955 she acquired the degree of the Doctor of Sciences. Straub’s later career turned towards science policy and politics. He became the member of the Hungarian Academy of Sciences in 1946 at an unusually young age of 32 and Full Professor in 1949 in Szeged. Later he was elected vice-president of the Academy for two periods and twice won the prestigious Kossuth Prize. He made essential contributions to the establishment of a separate biological section of the Hungarian Academy of Sciences, as well as to the foundation of the Biological Research Centre in Szeged in 1971. In later years, between 1988 and 1989, as President of the Presidential Council, he served as the president of Hungary. Detailed biograpies and resumés of the scientific achievements of Banga, Straub and Szent-Györgyi appeared recently (Hannus 2018; Hargittai 2018; Venetianer 2018).

## Closing remarks

The pioneering work by the Szent-Györgyi school laid the foundations of the modern era of muscle biochemistry and biophysics. Evidence of the significance of the discoveries has been borne out by the test of time, as the initial findings of the lab on the properties and behavior of actin, myosin and actomyosin were corroborated by subsequent structural and functional investigations. The discovery of actin opened a new avenue in science and inspired curiosity and research efforts that persist even today.

## Footnotes

1 The *Studies* is also available in the Archives of the Department of Biophysics, University of Pécs, Hungary with introductory notes provided by András Szent-Györgyi. http://actin.aok.pte.hu/archives/index.php.

2 Weber’s solution: 0.6 M KCl, 0.04 M NaHCO_3_, 0.01 M Na_2_CO_3._

3 Straub’s subsequent analysis of different conditions of actin preparation revealed that distilled water extraction of acetone powder resulted in an *“inactive form”* of the actin—corresponding to its monomeric form—that did not activate myosin (see the subsequent chapter). In fact, the actin obtained from the first preparations and used in the above mentioned viscometry experiments was filamentous actin that could *“activate”* myosin. Straub suspected that in his first preparations grinding or stirring before or during the extraction resulted in actin that is *„partly or mostly activated”*, i.e. polymerized. Straub (1943). Actin II, S. Karger Basel, New York; R. Gergely, Budapest.

4 https://www.nobelprize.org/prizes/medicine/1937/summary/.
